# Stevia Nonsweetener Fraction Displays an Insulinotropic Effect Involving Neurotransmission in Pancreatic Islets

**DOI:** 10.1155/2018/3189879

**Published:** 2018-04-29

**Authors:** Silvano Piovan, Audrei Pavanello, Giuliana Maria Ledesma Peixoto, Camila Cristina Ianoni Matiusso, Ana Maria Praxedes de Moraes, Isabela Peixoto Martins, Ananda Malta, Kesia Palma-Rigo, Claudinéia Conationi da Silva Franco, Paula Gimenez Milani, Antonio Sérgio Dacome, Silvio Claudio da Costa, Paulo Cezar de Freitas Mathias, Cecília Edna Mareze-Costa

**Affiliations:** ^1^Department of Physiology Sciences, Universidade Estadual de Maringá, Maringá, PR, Brazil; ^2^Department of Cell Biology and Genetics, Universidade Estadual de Maringá, Maringá, PR, Brazil; ^3^Department of Biochemistry, Universidade Estadual de Maringá, Maringá, PR, Brazil

## Abstract

*Stevia rebaudiana* (Bert.) Bertoni besides being a source of noncaloric sweeteners is also an important source of bioactive molecules. Many plant extracts, mostly obtained with ethyl acetate solvent, are rich in polyphenol compounds that present insulinotropic effects. To investigate whether the nonsweetener fraction, which is rich in phenolic compounds isolated from *Stevia rebaudiana* with the solvent ethyl acetate (EAF), has an insulinotropic effect, including interference at the terminals of the autonomic nervous system of the pancreatic islets of rats. Pancreatic islets were isolated from Wistar rats and incubated with EAF and inhibitory or stimulatory substances of insulin secretion, including cholinergic and adrenergic agonists and antagonists. EAF potentiates glucose-stimulated insulin secretion (GSIS) only in the presence of high glucose and calcium-dependent concentrations. EAF increased muscarinic insulinotropic effects in pancreatic islets, interfering with the muscarinic receptor subfamily M_3_. Adrenergic inhibitory effects on GSIS were attenuated in the presence of EAF, which interfered with the adrenergic *α*_2_ receptor. Results suggest that EAF isolated from stevia leaves is a potential therapy for treating type 2 diabetes mellitus by stimulating insulin secretion only in high glucose concentrations, enhancing parasympathetic signal transduction and inhibiting sympathetic signal transduction in beta cells.

## 1. Introduction

*Stevia rebaudiana* (Bert.) Bertoni is exploited worldwide for its leaves, which contain in diterpene glycosides with high sweetening power [1, 2] that is up to 300 times greater than that of sucrose [3]. Stevia sweeteners, which are nontoxic and nonmutagenic compounds [4], are an alternative to artificial sweeteners that, despite widespread use, are still of concern. Animal studies indicate that artificial sweeteners not only promote food intake and body weight gain but also induce metabolic changes that increase the risks of obesity, type 2 diabetes mellitus (DM2), and cardiovascular disease [5]. Stevia, however, does not cause cardiometabolic dysfunctions [6].

In addition to being a source of noncaloric sweeteners, stevia is also an important source of bioactive molecules. The results reported in the literature, in different experimental animal models as well as in humans, indicated that stevia has hypoglycemic properties [7–9], stimulating insulin secretion *in vitro* [10–12] and presenting antihyperglycemic, insulinotropic, and glucostatic effects [13, 14].

Glucose is the main physiological stimulant of insulin secretion in mammals [15]. When taken up by pancreatic beta cells by the GLUT2 isoform transporter, glucose is phosphorylated by the enzyme glucokinase to form glucose-6-phosphate [16]. Glucose-6-phosphate molecules can follow different pathways; however, glucose is primarily metabolized, increasing the ATP/ADP ratio and causing the inactivation of ATP-sensitive potassium channels (K_ATP_). The decrease in the conductance of potassium ions leads to depolarization of the cell membrane and consequently, the opening of voltage-dependent calcium channels. The increase in intracellular calcium causes exocytosis of the insulin granules [17]. In addition to the stimulus of insulin release by glucose, the beta cell is submitted to intense neural regulation.

The pancreatic islets are widely innervated by parasympathetic and sympathetic branches. Thus, catecholamines and acetylcholine (ACh) are considered signals of great importance for the regulation of pancreatic beta cell activity [18]. The autonomic parasympathetic nervous system regulates insulin secretion through the vagus nerve, which releases ACh into the neuronal junctions of the islets for binding to muscarinic cholinergic receptors [19]. While five subtypes of muscarinic receptors have been described in pancreatic beta cells (M_1_–M_5_), the subfamily M_3_ is crucial to the cholinergic insulinotropic effect [20]. The presence of *α*_2_ adrenergic receptors in pancreatic beta cells has also been reported, and these receptors have an inhibitory action on insulin secretion, which is mediated by epinephrine and norepinephrine. Both signals from the autonomic nervous system (ANS) are transduced through G proteins. Cholinergic signals cause stimulatory effects, and adrenergic signals cause inhibitory effects by G proteins. Although, ACh stimulates calcium influx, catecholamine inhibition of adenylyl cyclase causes suppression of calcium influx by voltage-dependent channels [21].

Many functional foods and herbal products have antioxidant substances and present insulinotropic effects. However, the mechanisms involved in improving insulin secretion and glycemia are not known. In view of the complexity of the process of modulating insulin secretion and the lack of studies showing the mechanisms by which the substances produced by stevia act on glycemic homeostasis, the objective of the present study was to test whether the fraction of stevia isolated with the solvent ethyl acetate (EAF), rich in phenolic compounds and low contamination of glycosides, interferes with insulin secretion stimulated by glucose in the presence or absence of ANS neurotransmitters.

## 2. Methods

### 2.1. Animals

The experimental procedures were approved by the Committee of Ethics in Animal Experimentation (CEUA-9076141116) of the State University of Maringá.

Male Wistar rats (*Rattus norvegicus*) were used, provided by the Central Animal House of the State University of Maringá. The animals were kept in boxes (4 animals/carton) of polypropylene (40 cm × 33 cm × 15 cm) at the Department of Physiological Sciences, under controlled conditions (23 ± 2°C and photoperiod of 12 hours light/12 hours dark). Standardized balanced “ad libitum” was fed to rats (Nuvilab CR1®, Nuvital, Colombo, PR).

### 2.2. Preparation and Composition of *Stevia rebaudiana* Fractions

The processes of extraction and fractionation of stevia leaves as well as analyses of proximal composition were carried out at the “Center for the Study of Natural Products” (NEPRON) at the State University of Maringá, from the *Stevia rebaudiana* leaves-seminal variety UEM 13, cultivated in the same institution. Several fractions were obtained from methanolic extracts, among them, ethyl acetate fraction (EAF), evaluated in this article, rich in phenolic compounds and also with a high value of antioxidant activity.

### 2.3. Proximal Composition and Identification of Phenolic Compounds of EAF

The proximal composition of EAF, its phenolic compounds, and low glycoside concentration were determined by means of an LC-MS/MS (Supplementary Material, Table S1) analysis. A chromatogram shows that the main phenolic compounds present in EAF are caffeic acid, quercetin 3-O-glycoside, cyanidin-3-glycoside, kaempferol, quercetin, apigenin, and rosmarinic acid (Supplementary material, Figure S1) [22].

### 2.4. Isolation of Pancreatic Islets

The pancreatic islets were isolated by the collagenase method [23]. Male rats, approximately 90 days old and overnight fasted for 12 hours, were anesthetized (thiopental 40 mg/kg + lidocaine 10 mg/kg, 0.6 ml/100 g p.c. i.p.) and then euthanized by decapitation. After median laparotomy, 8 mL of collagenase solution (collagenase type V—Sigma Chemical CO—0.7 mg/mL) was perfused via the bile duct/pancreatic duct in order to expand the pancreatic parenchyma. The pancreas was then removed, placed in a conical tube with collagenase solution, and incubated at 37°C for 15 minutes. With the aid of a stereomicroscope, the islets were collected, one by one, with an open-ended pipette. The isolated islets were preincubated with 5% CO_2_ and 95% O_2_ at 37°C for 60 minutes.

### 2.5. Islet Incubation

After preincubation, the solution was replaced with 1 mL Krebs/Ringer's solution containing EAF (0.3 *μ*g/mL) with 5.6 mM, 8.3 mM, or 16.7 mM glucose. The islets were incubated with 5% CO_2_ and 95% O_2_ at 37°C for 60 minutes. After this period, samples of the incubation medium were collected in test tubes and stored at −20°C for subsequent quantification of the secreted insulin.

In order to investigate how EAF stimulates insulin secretion, we first tested whether the observed effect is calcium dependent and the possible effects on ATP-sensitive potassium channels (K_ATP_). After preincubation, 0.3 *μ*g/mL of EAF in 16.7 mM glucose was added in the presence or absence of verapamil (50 *μ*M) [24] or diazoxide (250 *μ*M) [25], the former being a calcium channel blocker and the latter a K_ATP_ channel activator. It was also evaluated whether EAF causes membrane disruption using KCl (30 mM), a depolarizing agent [26].

Subsequently, experiments were performed to evaluate the participation of cholinergic and adrenergic receptors in insulin secretion induced by EAF. After preincubation, 0.3 *μ*g/mL of EAF was added in 8.3 mM glucose in the presence or absence of acetylcholine (ACh) (10 *μ*M) [27] and in the presence or absence of 4-diphenylacetoxy-N-methylpiperidine (4-DAMP) (100 *μ*M), an antagonist of muscarinic M_3_ receptor [28], or in the presence or not of metoctramine (1 *μ*M), an antagonist of muscarinic M_2_ receptor [20].

The presence of epinephrine (1 *μ*M) [27], as well as yohimbine (10 *μ*M) [29], an antagonist of adrenergic *α*_2_ receptor, and propranolol (1 *μ*M), an antagonist of adrenergic *β*_1_ and *β*_2_ receptors [30], were evaluated under the same incubation conditions (0.3 *μ*g/mL FAE in 16.7 mM glucose). All incubations were performed for 60 minutes at 37°C with 5% CO_2_ and 95% O_2_. Samples of the incubation medium were stored in a freezer at −20°C for further quantification of the secreted insulin.

### 2.6. Dosage of Insulin

The concentration of insulin in the incubation medium was determined by radioimmunoassay method. This method is based on the competition between ^125^I-labeled recombinant human insulin (PerkinElmer) and unlabeled insulin by the anti-insulin antibody (Sigma-Aldrich) produced in guinea pigs. Considering that the amounts of radioactive hormone and antibody are constant, complex formation (labeled insulin/antibody) depends on the amount of cold insulin present in the solution [31]. The intra- and interassay coefficients of variation were 12.2 and 9.8%, respectively, for insulin. The detection limit for the insulin level was 1.033 pmol/L. A gamma counter (Wizard2 Automatic Gamma Counter, TM-2470, PerkinElmer) was used to measure radioactivity.

### 2.7. Statistical Analysis

Data were submitted to analysis of variance (one-way ANOVA, Tukey) or Student's *t*-test with significance level when *p* < 0.05. The analyses were performed in the program GraphPad Prism version 6.0 (Windows GraphPad Prism Software, San Diego, CA, USA).

## 3. Results

**Figure 1:**
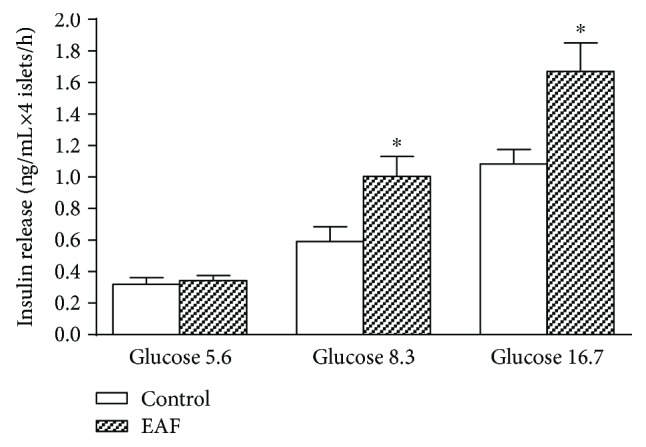
Effects of ethyl acetate fraction (EAF) (0.3 *μ*g/mL) on insulin secretion in pancreatic islets isolated from rats and incubated in medium containing 5.6, 8.3, or 16.7 mM glucose. The values represent the mean ± SEM (*n* = 20; ^∗^ *p* < 0.01 in relation to the control, *t*-test).

Figure 1 shows insulin secretion of isolated pancreatic islets in the presence and absence of EAF (0.3 *μ*g/mL) at different glucose concentrations. It was found that in both 8.3 and 16.7 mM, the compound significantly stimulated insulin secretion (*p* < 0.01), with no effect in the presence of 5.6 mM of the secretagogue.

**Figure 2:**
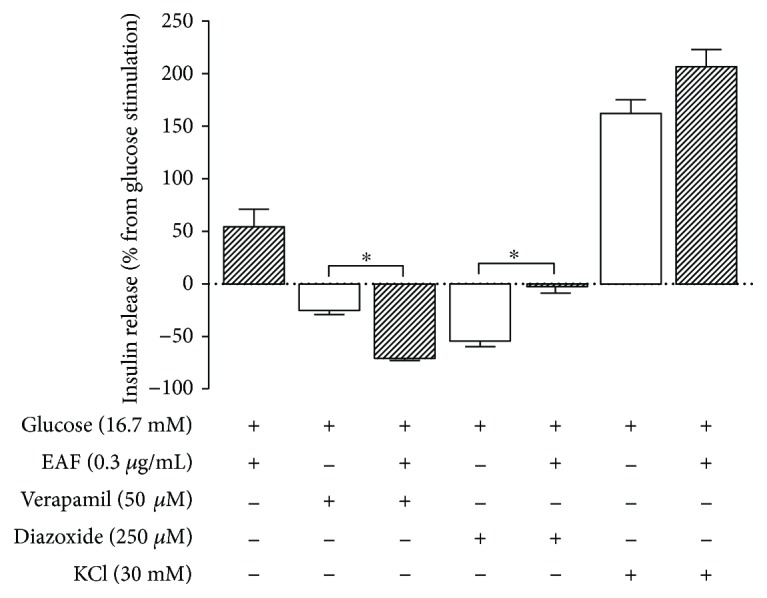
Effect of verapamil, diazoxide, and KCl on insulin secretion in the absence (empty bars) or presence (filled bars) of the ethyl acetate fraction (EAF) (0.3 *μ*g/mL). Insulin secretion was stimulated or inhibited as indicated above or below the *x*-axis, respectively. Values represent percentage (mean ± SEM; *n* = 20), calculated from insulin secretion in 16.7 mM glucose represented by line 0 (^∗^ *p* < 0.05 in relation to control, one-way ANOVA, Tukey).

The results presented in Figure 2 represent an increase or decrease in insulin secretion of isolated islets, expressed as percentages, calculated from the secretion values occurring in the presence of 16.7 mM glucose. It was found that the addition of EAF increased the glucose-stimulated insulin secretion (GSIS) by approximately 50% (*p* < 0.01). The reduction of GSIS caused by verapamil was significantly higher in islets incubated in the presence of EAF (70% reduction) than in the absence of EAF (25% reduction) (*p* < 0.01). Diazoxide, a K_ATP_ channel activator, had a significantly higher inhibitory effect on insulin secretion in the absence of EAF (50%) than in the presence of EAF (2%) (*p* < 0.01). EAF did not significantly alter GSIS in the presence of KCl, a depolarizing agent.

**Figure 3:**
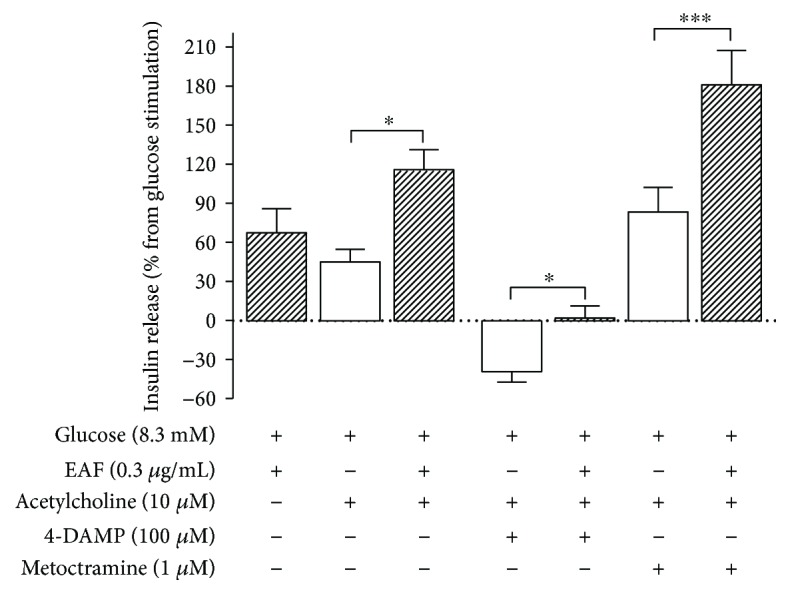
Effect of acetylcholine, 4-DAMP, and metoctramine on insulin secretion in the absence (empty bars) or presence (filled bars) of the ethyl acetate fraction (EAF—0.3 *μ*g/mL). Insulin secretion was stimulated or inhibited as indicated above or below the *x*-axis, respectively. Values represent a percentage (mean ± SEM; *n* = 20) of the insulin secretion in 8.3 mM glucose represented by line 0 (^∗^ *p* < 0.05; ^∗∗∗^ *p* < 0.001 in relation to control, one-way ANOVA, Tukey).

Figure 3 shows the results expressed as percentages, calculated from the insulin secretion values found in the presence of 8.3 mM glucose. The addition of ACh to the incubation medium increased GSIS by approximately 45%, and when added together with EAF, an additional 69% (*p* < 0.01) occurred, doubling GSIS values. 4-DAMP, an antagonist of the M_3_ receptor, reduced the GSIS by 38% (*p* < 0.01), but in the presence of EAF, this effect is not observed; methoctramine, an antagonist of the M_2_ receptor, caused an increase in GSIS of 83% and 180% in the absence and presence of EAF, respectively.

**Figure 4:**
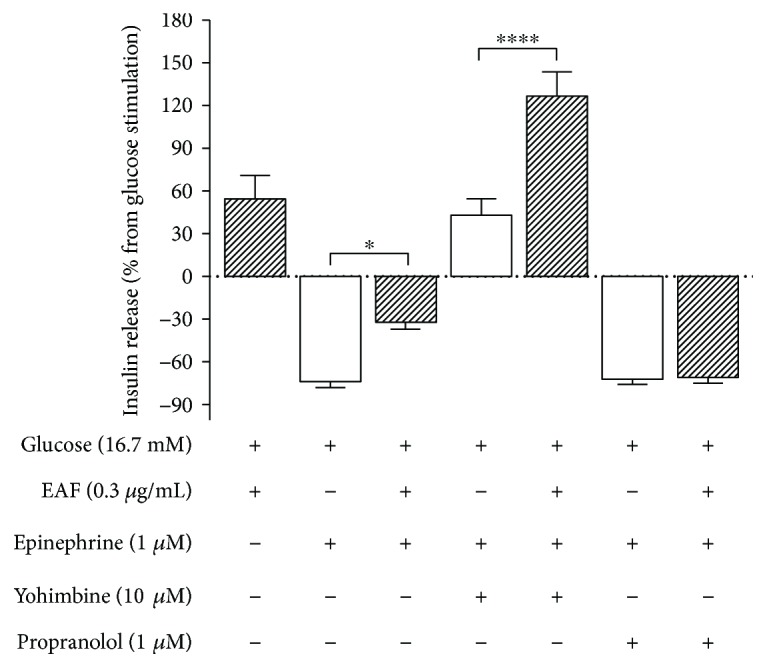
Effect of epinephrine, yohimbine, and propranolol on insulin secretion in the absence (empty bars) or presence (filled bars) of the ethyl acetate fraction ((EAF) 0.3 *μ*g/mL). Insulin secretion was stimulated or inhibited as indicated above or below the *x*-axis, respectively. The values represent percentage (mean ± SEM; *n* = 20) of the insulin secretion in 16.7 mM of glucose represented by line 0 (^∗^ *p* < 0.05; ^∗∗∗∗^ *p* < 0.0001 in relation to the control, one-way ANOVA, Tukey).

Figure 4 shows the results expressed as percentages of the increase or decrease of insulin secretion, having as a baseline the results obtained in the presence of 16.7 mM glucose. The presence of epinephrine reduced GSIS by almost 75%, whereas the presence of EAF in the incubation medium reduced this inhibitory effect by 32% (*p* < 0.01). Yohimbine, an antagonist of the *α*_2_ receptor, increased GSIS by 42%, and in the presence of EAF, this increase was approximately 3 times greater (*p* < 0.001). Both in the presence and absence of EAF, the reduction of GSIS caused by propranolol, an antagonist of *β*_1_ and *β*_2_ receptors, was approximately 70%.

## 4. Discussion

This is the first work that evaluates the effects on insulin secretion by a fraction of extracts of *Stevia rebaudiana* that is rich in phenolic compounds and nonsweetener. The most important result found in this study was the fact that EAF stimulates insulin secretion only in the presence of high glucose concentrations. This effect is extremely advantageous when, for example, compared with the effects of sulfonylurea, a drug widely used as an oral hypoglycemic drug that stimulates insulin secretion even at low glucose concentration and may, with prolonged use, lead to deterioration of type 2 diabetes control [32]. The antidiabetic sulfonylurea acts on K_ATP_ channels, inactivating them and depolarizing the membrane, which in turn leads to opening of the calcium channels and release of insulin granules. This mechanism operates at low or high concentrations of glucose [33]. A new generation of antidiabetics, such as those acting as incretinomimetics, has an advantage over sulfonylureas. As with GLP-1, these drugs act by inactivating the K_ATP_ channel at high glucose concentrations, but when glucose levels decrease, they may promote the opening of these channels by other mechanisms [34]. This duality of GLP-1 allows better control of insulin secretion and prevents beta cell overload. Similar to incretinomimetic substances, as shown in the current work, EAF showed insulinotropic effect sat high glucose concentrations but not at low glucose concentrations. As with incretinomimetic substances, EAF acts in postprandial and not in fasting situations.

Other studies have previously found that stevia has insulinotropic properties, but most of those studies refer to the glycoside sweetener or total extract of stevia leaves.

Extracts rich in polyphenol compounds from several plants have been reported to be effective against cell damage mediated by reactive oxygen species by increasing antioxidant defenses and reducing hyperglycemia [35]. Fractions obtained using the ethyl acetate solvent have a high concentration of polyphenols, as has been obtained from *Trichilia catigua* [36], grape skin [37], and stevia leaves [22, 38]. In addition, insulin-producing effects of polyphenols in chronic treatment *in vivo*, in pancreatic islet culture, and in insulin-producing cells [39, 40] have been reported, but direct effects on isolated islets, such as those shown in the current work with a fraction of stevia, have not been previously reported.

As previously mentioned, EAF stimulated GSIS only at high glucose concentrations. There are no *in vitro* studies showing the effects of antioxidant compounds on insulin secretion from pancreatic islets. However, it is known that the decrease in the formation of reactive oxygen species in these cells is directly related to the increase of GSIS [41].

To investigate the mechanisms involved in the increase of GSIS caused by EAF, a K_ATP_ channel enhancer and a voltage-dependent calcium channel blocker were used. Diazoxide is a compound that acts on the K_ATP_ channels, increasing the conductance of the potassium ion and causing hyperpolarization of the plasma membrane [42]. Our results indicate that the EAF components were able to maintain GSIS levels even in the presence of diazoxide. It has already been shown that molecules with antioxidant actions block the K_ATP_ channels [39]; therefore, it may be a possible mechanism involved in the action of EAF. However, the other results lead us to note that there may be no involvement of K_ATP_ channels. In addition, the rebaudioside A and stevioside glycosides, which also stimulate insulin secretion in the presence of high glucose concentrations, were not able to reverse the action of diazoxide on K_ATP_ channels [7, 9].

In the presence of verapamil, a voltage-dependent calcium channel blocker [43], EAF failed to stimulate GSIS, but when incubated with high KCl concentration, which acts as a depolarizing agent, there was no significant difference in GSIS. Such results suggest that EAF does not cause perturbation in beta cell depolarization and that its insulinotropic effect is critically dependent on extracellular Ca^2+^.

The muscarinic M_3_ receptor is the receptor subtype critical for cholinergic transduction in many cell types, such as in beta cells [44]. Muscarinic cholinergic agents or muscarinic cholinergic receptor antagonists prevent the effects of ACh by blocking its binding to cell receptors in the parasympathetic junctions [45]. The 4-DAMP compound binds to the muscarinic M_3_ receptor and blocks the effects of ACh on this receptor [46]. Our results showed that 4-DAMP significantly reduced the action of ACh on insulin secretion, but in the presence of EAF, its inhibitory effect was decreased. Therefore, these results indicate that a possible alteration in muscarinic activity is caused by stevia EAF via M_3_ receptors or its signaling pathway.

Among the 5 muscarinic subfamily receptors, binding of M_2_ and M_4_ (the even numbered receptors) with ACh reduces the insulinotropic effect caused by M_1_ and M_3_ (the odd numbered receptors) [20], indicating that a possible additive effect of EAF in GSIS does not involve M_2_ receptors or its signaling pathway.

It has been reported that plant extracts containing antioxidant properties interfere with muscarinic receptors, improving memory and contractile activity of the intestinal muscle [47]. In the present work, the results showed that cholinergic M_3_ receptors may be a possible target of the insulinotropic action of EAF.

We also investigated the role of adrenergic receptors. The inhibitory effects on GSIS caused by epinephrine were attenuated in the presence of EAF. The effect of yohimbine was significantly greater in the presence of EAF, suggesting that the adrenergic *α*_2_ receptor or the signaling promoted is a possible target of this fraction. Propranolol caused GSIS reduction, indicating that adrenergic *β* receptor blockade may provide a greater amount of epinephrine to *α*_2_ receptors, with a consequent inhibitory action.

In conclusion, the ethyl acetate fraction, isolated from leaves of *Stevia rebaudiana*, potentiated insulin secretion in the presence of high glucose concentrations. This effect may involve autonomic nervous system terminal activity. Taken together, the data obtained in the present work show that the compounds contained in EAF, without sweetening properties, may be a significant therapy for the treatment of DM2.

## Supplementary Materials

Supplementary Materials Table S1: proximal composition (g/100 g) of the EAF and LCMS identification of bioactive compounds. Figure S1: chromatogram for identification of the phenolic compounds of the *Stevia rebaudiana* ethyl acetate fraction (EAF) in LCMS and LC-MSMS. (1) Caffeic acid. (2) Quercetin 3-O-glycoside. (3) Cyanidin-3-glycoside. (4) Kaempferol. (5) Quercetin. (6) Apigenin. (7) Rosmarinic acid. Source: Milani et al. [22].
