# Supplementary material for: Stevia Nonsweetener Fraction Displays an Insulinotropic Effect Involving Neurotransmission in Pancreatic Islets
Source: Int J Endocrinol. 2018 Apr 29;2018:3189879. doi: 10.1155/2018/3189879 (PMC5949184; doi:10.1155/2018/3189879)
Supplement: Supplementary Materials — Table S1: proximal composition (g/100 g) of the EAF and LCMS identification of bioactive compounds. Figure S1: chromatogram for identification of the phenolic compounds of the Stevia rebaudiana ethyl acetate fraction (EAF) in LCMS and LC-MSMS. (1) Caffeic acid. (2) Quercetin 3-O-glycoside. (3) Cyanidin-3-glycoside. (4) Kaempferol. (5) Quercetin. (6) Apigenin. (7) Rosmarinic acid. Source: Milani et al. [22]. [file 3189879.f1.docx]

**SUPPLEMENTARY MATERIAL**

**Table S1.** Proximal composition (g / 100 g) of the ethyl acetate fraction (EAF) and LCMS identification of bioactive compounds.

| Composition | g/100g | |
| --- | --- | --- |
| Total Proteins | 34,5 ± 0,02* | |
| Total Lipids | 1,00 ± 0,03 | |
| Total Glycosides | 0,05 ± 0,01 | |
| Humidity | 3,04 ± 0,01 | |
| Fixed mineral residue | 2,24 ± 0,01 | |
| Flavonoids | 0,038 ± 0,01 | |
| Phenolic compounds | 52,42 ± 0,03 | |
| Compounds | RT | M/Z |
| Caffeic acid | 4.78 | 163 |
| Quercetin-3-o-glycoside | 4.88 | 434 |
| Cyanidin-3-glycoside | 4.51 | 449 |
| Kaempferol | 5.54 | 287 |
| Quercetin | 5.93 | 303 |
| Apigenin | 6.27 | 271 |
| Rosmarinic acid | 6.58 | 361 |
| Chlorogenic acid | 4.90 | 353 |
| Dicafeoylquinic acid | 5.33 | 515 |

RT = Retention Time; M / Z = mass. * Data express the mean ± e.p.m.

Source: Milani et al. (2017)


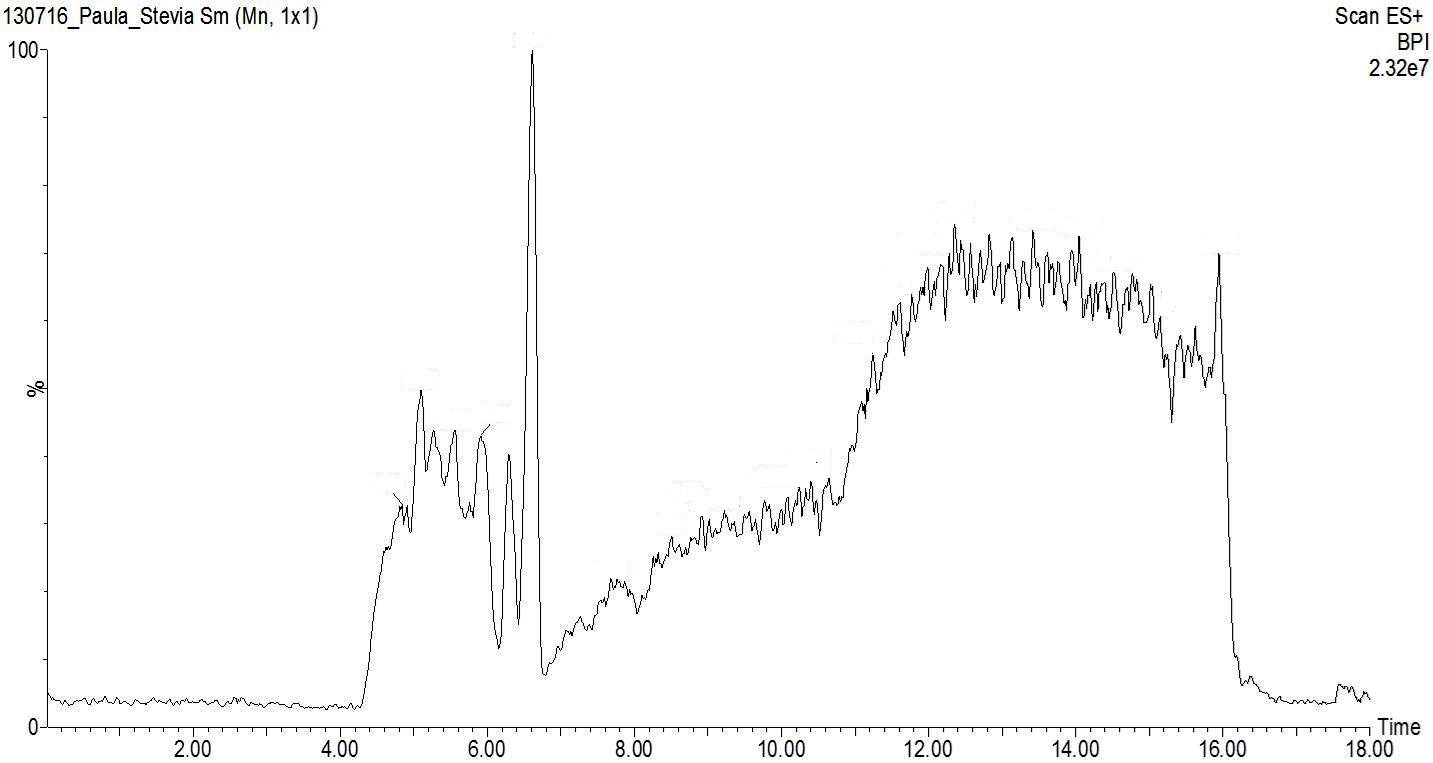


(2)

(3)

(4)

(6)

(1)

(5)

(7)

**Figure S1.** Chromatogram for identification of the phenolic compounds of the *Stevia rebaudiana* ethyl acetate fraction (EAF) in LCMS and LC-MS / MS. (1) Caffeic acid. (2) Quercetin-3-o-glycoside. (3) Cyanidin-3-glycoside. (4) Kaempferol. (5) Quercetin. (6) Apigenin. (7) Rosmarinic acid.

Source: Milani et al (2017).
